# Cutaneous manifestations in patients with end-stage renal disease on hemodialysis

**DOI:** 10.22088/cjim.14.4.728

**Published:** 2023

**Authors:** Azar Shirzadian Kebria, Zeinab Aryanian, Amin Choobdar, Roghayeh Akbari

**Affiliations:** 1 Department of Dermatology, Babol University of Medical Sciences, Babol, Iran; 2 Bullous Diseases Research Center, Tehran University of Medical Sciences, Tehran, Iran; 3 Department of Nephrology, Babol University of Medical Sciences, Babol, Iran

**Keywords:** Cutaneous manifestation, Hemodialysis, End-stage renal disease

## Abstract

**Background:**

End-stage renal disease (ESRD) is a serious chronic disease that affects many organ systems. Skin manifestations that are commonly seen in ESRD can significantly impair the quality of life in these patients. Early recognition and management of mucocutaneous disorders can improve quality of life and decrease morbidity. This study aimed to evaluate the skin manifestations in ESRD patients undergoing hemodialysis.

**Methods:**

In this cross-sectional study 150 patients undergoing hemodialysis in the Nephrology Department of Shahid-Beheshti Hospital in Babol were enrolled. The demographic and clinical data were assessed. Analysis was done by SPSS 22 and significance level was under 0.05.

**Results:**

The mean duration of hemodialysis was 8.7 months. The most common skin findings in patients include xerosis 84.7%, pallor 82.7%, pruritus 67.3%, hyperpigmentation 40%, purpura 28%. Skin infections were detected in 36% of patients (fungal 28%, bacterial 10.7%, and viral 5.3%). Nail, hair and mucosal changes were observed among 65.3%, 38% and 17.3% of patients respectively. No significant correlation was detected between skin findings and duration of dialysis.

**Conclusion:**

The findings of the present study showed that skin manifestations are highly prevalent among patients with ESRD. Prompt diagnosis and management of the dermatological disorders may improve the quality of life in the affected patients.

End-stage renal disease (ESRD) is an important medical problem that its prevalence rate has increased over the years. It is considered to be caused by various diseases such as diabetes, hypertension, obstructive uropathy, acquired and congenital cystic disorders of the kidneys, glomerulonephritis, urinary tract infections and vasculitis (1). Hemodialysis is a treatment that replaces kidney function. Patients with end stage renal failure commonly exhibit various cutaneous changes that precede or follow initiation of dialysis treatment (2, 3). Skin changes in hemodialysis patients are mainly classified as specific skin manifestations (acquired perforating disorders (APD), bullous dermatoses, calcifying disorders, and nephrogenic fibrosing dermopathy) and non-specific skin manifestations (pruritus, xerosis, pallor, purpura, mucosal changes, nail and hair lesions and pigmentary changes) (4). Evidently, cutaneous involvement is prevalent in patients with ESRD, but the prevalence rate of various skin manifestations in these patients is different (2-9), that may be influenced by factors such as race, nutrition, socioeconomic condition of patients, climate, etc. Mucocutaneous disorders can be extensive and have a considerable negative effect on patients` quality of life. Early recognition of cutaneous signs and prompt treatment can reduce patients` morbidity and medical cost (5). Further acquaintance of dermatologists and other clinicians with dermatologic manifestations of ESRD may facilitate earlier detection and treatment of the disorders (2-4).

This study was designed to evaluate the frequency of various mucocutaneous manifestation in patients undergoing hemodialysis in the Nephrology Department of Shahid Beheshti Hospital in Babol.

## Methods

A cross-sectional study was conducted consecutively in 150 patients with ESRD undergoing hemodialysis who attended the Nephrology Department of Shahid Beheshti Hospital in Babol from March 2020 to March 2021. Ethical approval was obtained from the Research Ethics Committee (Ethical code: IR.MUBABOL.REC.1399.145) at Babol University of Medical Sciences; and written informed consent was recorded for each patient. Patients with concomitant primary skin disease and patients taking drugs that cause skin complications were excluded.

 A brief medical history, quality of any dermatological problems, demographic characteristics of patients such as sex, age, duration and cause of ESRD and duration of hemodialysis were recorded in the relevant checklist. All study participants underwent clinical examination. Dermatological manifestations were evaluated and confirmed by a dermatologist. Microscopic and histopathological evaluation was performed if needed. Potassium hydroxide examination was used for the diagnosis of dermatophytosis. The clinical diagnosis of Kyrle’s disease was confirmed by histopathological evaluation. The collected data were analyzed in SPSS Version 22. We compared the dermatological manifestations by Pearson’s chi-square test. A *p *< 0.05 was considered statistically significant.

## Results

One-hundred fifty dialysis patients were assessed in this study. Among them, 94 (62.7%) were males and 56 (37.3%) were females (male to female ratio 1.7:1). The mean age of the patients was 59 years. The age of the patients ranged from 15 years to 76 years. Majority of patients belonged to 40- 60 years’ age group. 

The mean duration of dialysis was 8.7 months. The underlying disease resulting in renal failure was diabetes (48.7%), hypertension (38.6%), Chronic glomerulonephritis (2.7%), others (7.3%), and indeterminate (2.7%) (table1).

**Table 1:** Frequency distribution of demographic data in the studied patients

**Variable**		**N**	**Percentage (%)**
**Age(years)**	<40	23	15.3
	40-60	70	46.7
	>60	57	38
**Sex**	Male	94	62.7
	Female	56	37.3
**Duration of dialysis** **(Months)**	<6	40	26.7
	6-12	83	55.3
	>12	27	18
**Underlying disease**	Diabetes	73	48.7
	Hypertension	58	38.6
	Chronic glomerulonephritis	4	2.7
	Others	11	7.3
	Indeterminate	4	2.7

All our patients had at least one associated mucocutaneous change. The most common skin findings in patients include xerosis 84.7%, pallor 82.7%, pruritus 67.3%, hyperpigmentation 40%, and purpura 28%. Sixty-six cutaneous infections (42 fungal, 16 bacterial, and 8 viral) were found among fifty-four patients in this study. Nail changes included discoloration (43.3%), onychomycosis (24.6%), half and half nail (6%) and others (4%). Hair changes noted in the study were scalp and body hair loss and discoloration. Xerostomia, glossitis, angular cheilitis and gingivitis were the common mucosal findings in study papulation (table 2). Kyrle’s disease was found in 2(1.3%). The cause of ESRD in both patients was diabetic nephropathy (table 2). Other specific cutaneous manifestations such as nephrogenic systemic fibrosis, calcifying disorders and bullous diseases were not found in our patients. In the current study, there was no significant correlation between skin manifestation and the duration of hemodialysis and no significant correlation was detected between skin changes and the cause of renal failure either. 

**Table 2:** Frequency distribution of cutaneous manifestations in the studied patients

**Variable**		**N**	**Percentage (%)**
**Pruritus**		101	67.3
**Pallor**		124	82.7
**Xerosis**		127	84.7
**Purpura**		42	28
**Hyperpigmentation**		60	40
**Skin infections**	Fungal	42	28
	Bacterial	16	10.7
	Viral	8	5.3
**Kyrle’s disease**		2	1.3
**Nail changes**	discoloration	65	43.3
	Onychomycosis	37	24.6
	Half and Half nail	9	6
	Others	6	4
**Hair changes**	Sparseness	34	22.7
	discoloration	27	18
	Others	8	5.3
**Mucosal changes**	Xerostomia	17	11.3
	Glossitis	14	9.3
	Angular cheilitis	8	5.3
	Gingivitis	5	3.3
	Others	8	5.3
**Others**		16	10.7

## Discussion

ESRD patients have various cutaneous manifestations that are associated with impaired quality of life. Accurate assessment and management of skin disorders can reduce morbidity of these patients (5). In this study, all of our patients showed one or more dermatological disorders. Some authors similarly reported at least one related skin change in their patients (2, 6-10). Xerosis was the most common skin disorders observed in 84.7% of our patients. Extremities especially lower limbs were the most affected sites. The prevalence of xerosis was estimated between 50% and 85% in other series (4, 5, 10). The xerosis is ascribed to diuretic use, atrophy of sweat glands and sebaceous glands and impaired integrity of stratum corneum in consequence of decreased water amount (11).

Another prevalent cutaneous manifestation in current study was pallor which was consistent with the findings of the study Anees et al. and Raiesifar et al. (12, 13). The frequency of 64% and 60% for pallor was reported by Sahadevan and Udayakumar respectively (2, 10). Paleness in dialysis patients can be ascribed to anemia due to decreased renal erythropoietin production as well as blood loss during dialysis (4).

Pruritus was detected in 67.3% of our patients. The prevalence of pruritus ranged between 50% and 90% in different studies (14). The pathogenesis of pruritus in ESRD patients is not exactly known, but factors such as uremia, hyperparathyroidism, neuropathy and increased levels of calcium and phosphorus and increased sensitivity to the compounds used in dialysis are considered (4). Forty percent of our patients had hyperpigmentation. A prevalence range of 20% to 50% was reported in the literature (14). Excessive amount of melanocyte stimulating hormone due to defective renal excretion could be attributed to diffuse hyperpigmentation in ESRD patients (4).

Cutaneous infections were found in 36% of patients. The fungal infections (28%) were the most common of them. Udayakumar et al. have reported a frequency of 30% while Bencicni et al. have noted a more prevalent result (67%) (2,15). The high prevalence of fungal infection in ESRD patients could be related to impaired function of T and B lymphocyte, diminished activity of natural killer cell and impaired phagocytosis (16). Hair, nail and mucosal lesions in our study were comparable with other studies. In this study, there was no relation between the duration of hemodialysis and the prevalence of skin changes in patients; these results were consistent with those of Hajheydari et al. and Mourad et al. (9, 17). In contrast with our study, Raiesifar et al. reported a significant relationship between duration of dialysis and skin disorders (13). 

All the 150 patients studied, displayed at least one cutaneous manifestation. Skin abnormalities in ESRD are common and various. With increased life expectancy in these patients, there is more possibility that newer skin abnormalities will be developed. Xerosis, pallor, pruritus and hyperpigmentation were the most frequent symptoms in our study. Early recognition and appropriate management of cutaneous disorders can lead to improved quality of life in these patients. 
